# Incorporation of reduced graphene oxide into faceted flower-like {001} TiO_2_ for enhanced photocatalytic activity

**DOI:** 10.1098/rsos.180613

**Published:** 2018-08-15

**Authors:** Haijin Liu, Peiyao Li, Haokun Bai, Cuiwei Du, Dandan Wei, Yuzhao Su, Yuqian Wang, Lin Yang

**Affiliations:** 1 School of Environmental Science, Key Laboratory for Yellow River and Huaihe River Water Environment and Pollution Control, Ministry of Education, Henan Key Laboratory for Environmental Pollution Control, Henan Normal University, Xinxiang 453007, People's Republic of China; 2 Faculty of Chemical, Environmental and Biological Science and Technology, Dalian University of Technology, Dalian 116024, People's Republic of China

**Keywords:** graphene, TiO_2_ (001), photocatalytic activity, visible light

## Abstract

Anatase TiO_2_ with {001} facets is much more active than that with {101} facets, which has been verified via experiments and theoretical calculations. Graphene has garnered much attention since it was initially synthesized, due to its unique properties. In this study, reduced graphene oxide (RGO)/{001} faceted TiO_2_ composites were fabricated via a solvothermal method. The composites were characterized by scanning electron microscopy, transmission electron microscopy, X-ray diffraction, X-ray photoelectron spectrophotometry, photoluminescence and Raman analysis. The results revealed that the graphene oxide was reduced during the preparation process of the {001} faceted TiO_2_, and combined with the surface of {001} TiO_2_. The photocatalytic activities of the composites were evaluated through the degradation of basic violet, under both white light (*λ* > 390 nm) and visible light (*λ* = 420 nm) irradiation. The results indicated that the photocatalytic activities of the {001} faceted TiO_2_ were significantly improved following the incorporation of RGO, particularly under visible light irradiation. Theoretical calculations showed that the band structure of the {001} faceted TiO_2_ was modified via graphene hybridization, where the separation of photoinduced electron–hole pairs was promoted; thus, the photocatalytic activity was enhanced.

## 1. Introduction

Semiconductor materials have been widely used in environmental remediation and solar conversion applications. Among those, TiO_2_ has attracted much attention due to its non-toxicity, biochemical stability, strong oxidative properties, low cost and photo-erosion resistance. However, the application of TiO_2_ has been restricted due to its low photo-conversion efficiency and limited light response range. Much research has been conducted towards improving the photocatalytic activities of TiO_2_, such as noble metal deposition, surface modification, metal and non-metal doping, and semiconductor coupling [1–12]. Several studies have shown that the properties of nanomaterials are intimately associated with their morphologies, dimensions, structures as well as exposed facets [13]. Further, it has been verified via theoretical calculations and experiments, that anatase TiO_2_ with {001} facets is much more active than that with {101} facets (which is thermodynamically stable). The average surface energy is as follows: 0.90 J m^−2^ {001} > 0.53 J m^−2^ {100} > 0.44 J m^−2^ {101} [14]. Although {001} facets possess a higher surface energy, which translates to increased chemical activity, anatase TiO_2_ is typically reported to contain {101} facets, and to a lesser degree, {100} facets [15].

Recently, many researchers have focused on the synthesis of {001} faceted TiO_2_. Wang *et al*. [16] theoretically and experimentally demonstrated the dual roles of HF in mediating crystal facet growth. At low concentrations, through surface fluorination, HF serves as a stabilizer (through surface fluorination) to facilitate the growth of the {001} facets, and preserve the grown {001} faceted surface. However, at high concentrations, HF selectively destroys the {001} faceted surface through –OH replacement and –TiOF_2_ dissolution processes. Han *et al*. [17] synthesized rectangular TiO_2_ nanosheets with highly reactive {001} facets as the top and bottom surfaces through a simple hydrothermal route. Such TiO_2_ nanosheets showed excellent photocatalytic efficiency due to the exposure of a high percentage of the {001} facets. Li *et al*. [18] obtained hierarchical flower-like TiO_2_ nanostructures that were dominated by {001} facets using a one-pot, template-free method. Further, these nanostructures exhibited higher photocatalytic activity for the degradation of methylene blue under ultraviolet–visible light irradiation. Zheng *et al*. [19] synthesized anatase TiO_2_ nanocrystals with a percentage of tunable reactive {001} facets via a microwave-assisted hydrothermal treatment. Their study revealed that both the exposed facets and surface chemistry played important roles in the photocatalytic activity of the anatase TiO_2_ nanocrystals.

In order to improve its photocatalytic efficiency, research has also been conducted on combining {001} faceted TiO_2_ with other materials. Zhang *et al*. [20] employed anatase TiO_2_ {001} facets as a substrate of a two-dimensional MoS_2_ composite, and compared the photocatalytic activities. The results showed that the MoS_2_/TiO_2_(001) composite with an optimal ratio demonstrated a significant enhancement in photocatalytic activity, up to 3.4 times that of pristine TiO_2_ nanosheets with exposed {001} facets. Yu *et al*. [21] synthesized self-doped carbon TiO_2_ sheets with exposed {001} facets via a hydrothermal method. These materials exhibited an enhanced absorption across the entire visible light region, an obvious red shift at the absorption edges, as well as much higher photocatalytic activity than that of C-doped TiO_2_ nanoparticles, due to the presence of exposed {001} facets.

Graphene is a two-dimensional (2D) carbon material with unique properties, including excellent conductivity and mechanical strength, high flexibility and a large surface area; hence, it has attracted considerable attention since it was initially fabricated. Owing to its unique properties, several researchers have attempted to introduce graphene to modify TiO_2_ towards the improvement of its photocatalytic activity [22,23].

Pan *et al*. [24] fabricated graphene–TiO_2_ nanowire (GNW) and graphene–TiO_2_ nanoparticle (GNP) nanocomposites, and found that GNW and GNP exhibited higher performance than TiO_2_ on its own, through the incorporation of graphene. Pant *et al*. [25] immobilized TiO_2_ nanofibres onto reduced graphene oxide (RGO) sheets by electrospinning and calcination. The synthesized TiO_2_/RGO composite revealed a remarkable increase in photocatalytic activity in contrast with pristine TiO_2_ nanofibres. Rahimi *et al*. [26] synthesized a novel heterogeneous photocatalytic TiO_2_–graphene nanocomposite system that was sensitized with tetrakis(4-carboxyphenyl)porphyrin, for the disinfection of bacteria in wastewater. Photocurrent measurements demonstrated that the TiO_2_–graphene nanocomposite, with 3% graphene content, had a higher photoactivity capacity. Yadav & Kim [27] prepared anatase TiO_2_–graphene oxide (GO) nanocomposites with different GO loadings by a solvothermal method. The TiO_2_–GO nanocomposites exhibited enhanced photocatalytic performance over pure TiO_2_ nanoparticles, in the degradation of gaseous benzene under UV light irradiation. In addition, a few of studies on graphene/{001} faceted TiO_2_ composites have been published [28–31]. The graphene/{001} faceted TiO_2_ or graphene/doped {001} faceted TiO_2_ composites exhibited enhanced photocatalytic activities towards the photocatalytic degradation of dyes [28,29], H_2_ production [30], CO_2_ reduction [31] and more. The enhanced photocatalytic performance of the graphene/{001} faceted TiO_2_ composites was attributed to the effective charge anti-recombination of graphene, high absorption in visible light region and the high catalytic activity of the {001} facets [28,31].

In this study, RGO/{001} faceted TiO_2_ was synthesized via a solvothermal method using water and ethanol as solvents. The effects of graphene loading on the photocatalytic activity of TiO_2_ were investigated using basic violet (BV) as model pollutant. The results indicated that RGO/{001} faceted TiO_2_ exhibited higher photocatalytic activity than that of pure {001} faceted TiO_2_ under both white and visible light irradiation.

## 2. Experimental procedure

### 2.1. Materials

Sodium nitrate (NaNO_3_, CAS no. 7631-99-4), sulfuric acid (H_2_SO_4_, CAS no. 7664-93-9), potassium permanganate (KMnO_4_, CAS no. 7722-64-7), hydrochloric acid (HCl, CAS no. 7647-01-1) and titanium tetrafluoride (TiF_4_, CAS no. 7783-63-3) were purchased from Shanghai Aladdin Biochemical Technology Co. Ltd (Shanghai, China). All chemicals were analytical-grade reagents and used as purchased without further purification.

### 2.2. Preparation of graphene oxide (modified Hummers method)

The slow addition of 1.0 g of graphite and 0.5 g of NaNO_3_ into 23.0 ml of concentrated H_2_SO_4_ proceeded in an ice–water bath. After stirring until completely dissolved, 3.0 g of KMnO_4_ was added into the mixture at 20°C. The obtained dark green reaction mixture was then heated to 30°C under continuous stirring for 30 min. Subsequently, 46.0 ml of deionized water was added dropwise, where the temperature was increased to 96°C and maintained for 15 min. Finally, 10.0 ml of H_2_O_2_ and 140.0 ml of deionized water were added to cease the reaction following 15 min of stirring. This mixture was then centrifuged, rinsed with distilled water and ethanol, and dried at 60°C.

### 2.3. Preparation of reduced graphene oxide/{001} faceted TiO_2_ composites

**Figure 1.**
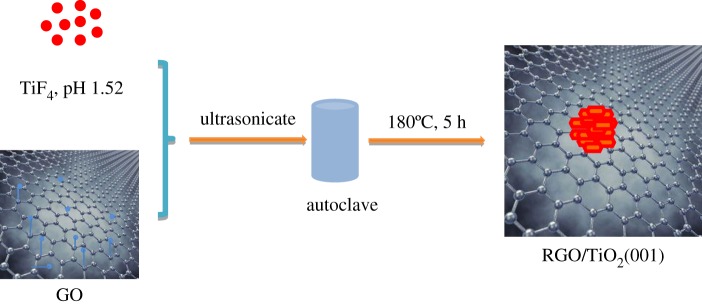
Illustration of preparation of RGO/{001} faceted TiO_2_.

Hydrochloric acid (1.5 M) was used to adjust the pH of deionized water (1.0 l) to approximately pH 2.0 and designated as solution ‘A’. Next, 0.563 g of TiF_4_ was added into 100 ml of solution A to obtain a 4.5 × 10^−2^ mM TiF_4_ stock solution. Solution ‘B’ was prepared by adding 5.0 ml of TiF_4_ stock solution into solution A and diluted to 50.0 ml. Solution ‘C’ was obtained by adjusting the pH of solution B to 1.52 using 0.1 M of an HCl solution. A certain volume of GO was dispersed into solution C by ultrasonication for 30 min, followed by transferal into a Teflon-lined autoclave, and maintained at 180°C for 15 h in an oven. When naturally cooled to room temperature, the composites were processed by filtration, rinsed with water and ethanol several times, and then dried at 60°C for 24 h. The complete process is illustrated in figure 1.

During the preparation of solution B, the composition of solution A was sometimes modified through the addition of 10 ml of ethanol, and changed amounts of GO, in order to obtain graphene/{001} TiO_2_ composites with different GO loadings (0, 5, 10, 15, 20%). The as-prepared composites were denoted as ET0–ET20 (with ethanol) and WT0–WT20 (without ethanol), respectively, where the numbers 0–20 represent the GO amount.

### 2.4. Synthesis of reduced graphene oxide

The RGO prepared for comparative studies was obtained according to the same procedure as for the preparation of RGO/{001} TiO_2_ composites without TiF_4_.

### 2.5. Characterization

The crystal structure was elucidated by X-ray diffraction (XRD) using a D8 Advance diffractometer (Bruker Company, Germany) with Cu K*α* (*λ* = 0.15406 Å) radiation, operated at 25 mA and 35 kV, and scanned between 10 and 80° at a speed of 0.02°/0.4. The morphology of the materials was observed with JSM-6390LV scanning electron microscopy (SEM) and JEM-2100 transmission electron microscopy (TEM) instruments (Japan). A Horiba LabRAMR HR-800 Raman spectrometer was employed to obtain Raman spectra of the materials with an excitation laser wavelength of 633 nm, and a 1800 l mm^−1^ grating, scanned in the range of 90 to 2000 cm^−1^ at a speed of 0.5 s^−1^. The chemical states and atomic surface elements of the materials were measured with a XSAM-800 X-ray photoelectron spectrophotometer (XPS). All binding energies were calibrated by taking the C 1s peak as a reference at 284.6 eV of contaminant carbon. The photoluminescence (PL) spectra were recorded with a florescence spectrophotometer (Cary Eclipse, Varian, USA) with a xenon lamp as an excitation source at an excitation wavelength of 340 nm.

### 2.6. Photocatalytic activity

The photocatalytic activities of the materials were quantified with a PCX50B Discover multichannel photoreactor (Perfect Light Technology Ltd) using BV as a model pollutant. The quartz reaction bottles were on the upper portion of the photoreactor and moved around at regular interval to guarantee uniform light irradiation on each bottle. Light-emitting diode (LED) light sources (*λ* > 390 nm, light intensity of 2.5 × 10^6^ lux) were installed beneath the bottles and remained in place under the bottles during the reactions. Nine experiments could be carried out in parallel/simultaneously, where there was no difference in light among the nine bottles. The light sources were cooled down during the reaction via the application of cold air. A controller was located at the bottom of the photoreactor to adjust the light sources, the magnetic stirring speed, bottle transfer as well as the reaction time.

The photocatalytic experiments were carried out as follows: 50 ml of BV solution (10 mg l^−1^) was introduced into a quartz bottle, and 10 mg of photocatalyst was added and stirred at 300 r.p.m. for 60 min in order to obtain an adsorption–desorption balance. Subsequently, the light was turned on to initiate the reaction, whereafter the quartz bottles were moved at 30 s intervals. A 4.0 ml volume of reaction solution was withdrawn every 30 min and immediately centrifuged at a speed of 10 000 r.p.m. for 10 min. The supernatant was then measured with a TU-1900 UV–visible spectrometer at a wavelength of 582 nm. Within 0–20 mg l^−1^, the relationship between the absorbance and BV concentration obeyed the Beer–Lambert law; hence, the degradation rate (*r*) of BV during the reaction could be calculated according to

$$r=\frac{c_{0}-c}{c_{0}}=\frac{A_{0}^{}-A}{A_{0}},$$

where *C*_0_ or *A*_0_ is the initial concentration or absorbance of the BV solution, *C* or *A* the concentration or absorbance of the BV solution during the photocatalytic reaction.

## 3. Results and discussion

### 3.1. X-ray diffraction analysis

**Figure 2.**
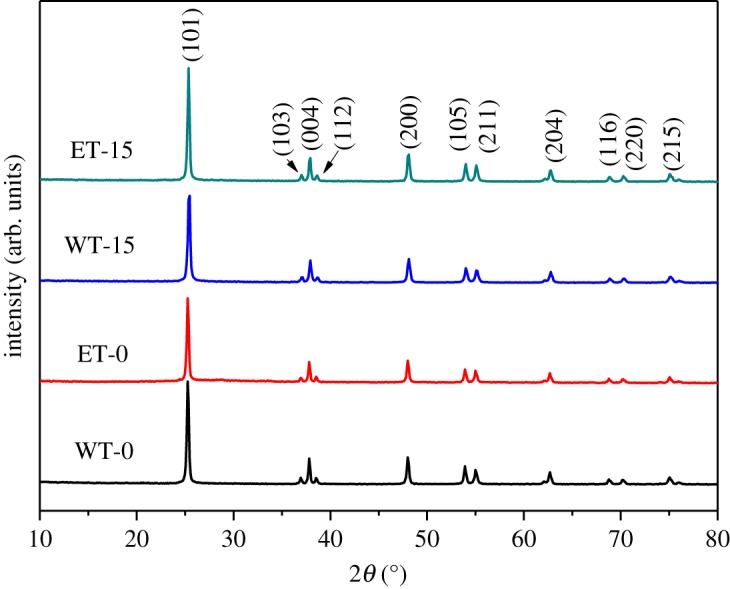
XRD patterns.

XRD was carried out to investigate the crystalline structure and composition of the samples. The XRD patterns of ET-15, WT-15, ET-0 and WT-0 are displayed in figure 2. It may be seen that diffraction peaks were assigned to (101), (004), (200), (105), (211), (204), (116), (220) and (215) faces. All peaks were consistent with those of JCPDS no. 21-1272, showing that the as-prepared samples were tetragonal. It was obvious that the peak positions of WT-15 and ET-15 did not change in contrast with those of WT-0 and ET-0, which indicated that the crystalline structure of TiO_2_ was not altered when combined with graphene. No graphene assigned peak was observed in the XRD pattern, which may have two causes:

i. The diffraction peak of graphene is located at 24.5°, which was very likely covered by the strong anatase peak at 25.3°.
ii. The low diffraction intensity and low concentration of graphene [32].

The particle sizes of the as-prepared materials could be calculated by the Scherrer equation:

$$D=\frac{k\lambda }{\beta \text{cos}\theta \;},$$

where *λ* is the X-ray wavelength; *β* the full width at the half maximum of the (101) peak; *θ* the incident angle; and *k* a constant, typically 0.89. Furthermore, two peaks located at 25.3° (101) and 48.2° (200) were selected to calculate the lattice constants, with the results displayed in table 1. It may be seen that compared to non-carbon-containing materials, the particle sizes of the carbon-containing materials decreased dramatically following the introduction of C, revealing that it significantly inhibited particle growth. Furthermore, the lattice constants were also altered by the introduction of C. The length of the *c* axis was obviously decreased in WT-15 and ET-15, in contrast with WT-0 and ET-0.

**Table 1.** Particle sizes and lattice constants.

sample	particle size (nm)	lattice constants	
		*a* = *b*	*c*
WT-0	23.7	3.7892	9.5398
ET-0	23.5	3.7836	9.6939
WT-15	21.1	3.7813	9.3406
ET-15	22.4	3.7857	9.2968

### 3.2. Scanning electron microscopy analysis

**Figure 3.**
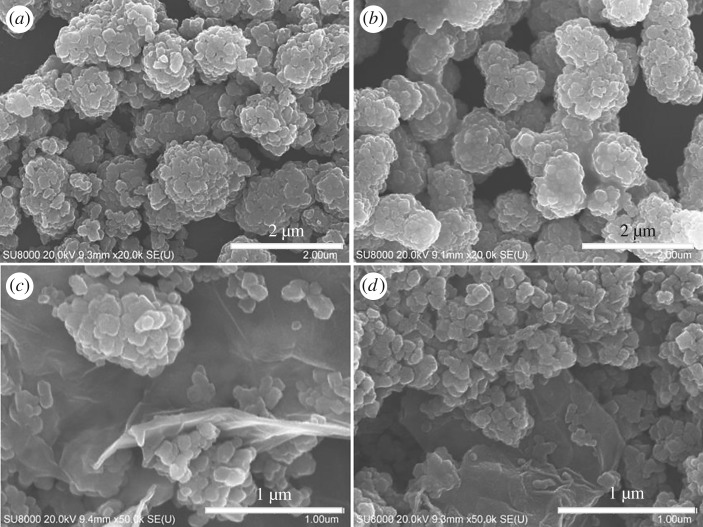
FESEM images of WT-0 (*a*), ET-0 (*b*), WT-15 (*c*) and ET-15 (*d*).

FESEM images of the samples are displayed in figure 3. A number of flower-like TiO_2_ products may be observed in figure 3*a* (WT-0), which comprised numerous nanosheets. The flower-like TiO_2_ products were uniform with size of about 1 µm. Figure 3*b* is the FESEM image of ET-0. Compared to WT-0, the flower-like TiO_2_ products were more uniform, and the nanosheet aggregates adhered to each other more tightly, indicating that the addition of alcohol during preparation process increased the stickiness of the nanosheets. The FESEM images of WT-15 and ET-15 are shown in figure 3*c,d*. It may be clearly seen that the flower-like TiO_2_ were scattered onto the thin surfaces of the graphene. Compared to WT-0 and ET-0, the dimensions of the flower-like TiO_2_ were slightly decreased.

### 3.3. Transmission electron microscopy analysis

**Figure 4.**
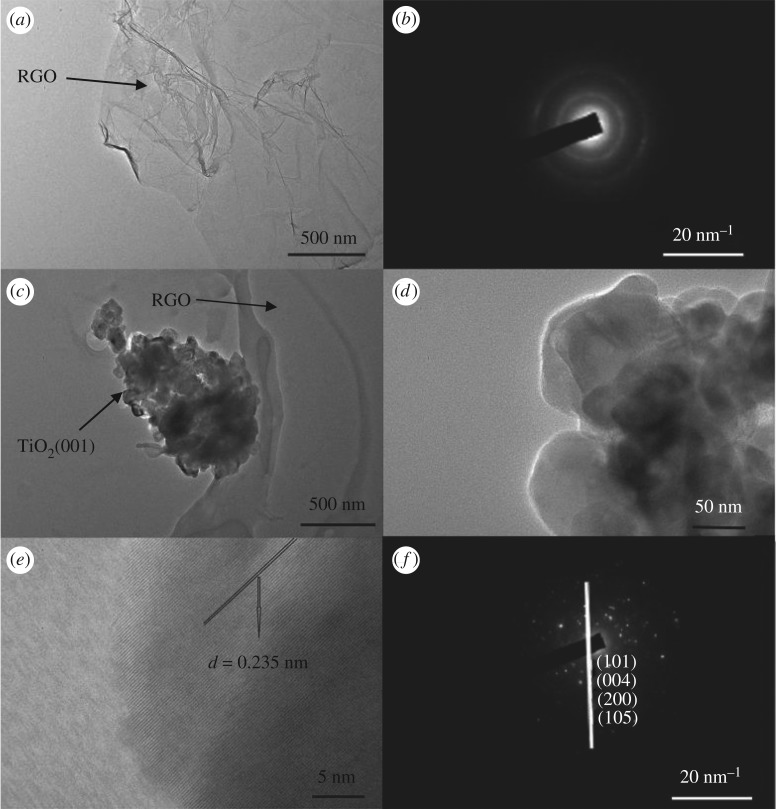
TEM image of RGO (*a*), SAED of RGO (*b*), TEM images of ET-15 (*c–e*) and SAED of ET-15 (*f*).

During the preparation of the {001} faceted TiO_2_, GO was reduced to RGO. The TEM image of the RGO is displayed in figure 4*a*. It can be seen that RGO was a transparent 2D thin sheet with several folds, showing that the GOs were split into flake structures and reduced to RGO. The selected area electron diffraction (SAED) image of the RGO (figure 4*b*) was composed of two rings. The inner ring was extremely bright, which was assigned to the (1100) face, whereas the outer ring was assigned to the (0001) face, indicating that the sample was an ultrathin graphene flake [33]. Figure 4*c,d* shows TEM images of ET-15. It may be observed that the TiO_2_ was attached to the surface of the graphene, and the TiO_2_ structure consisted of many nanosheets, which were approximately 50 nm in size. The lattice fringes of the small nanosheets may be clearly seen in figure 4*e*, which were approximately 0.235 nm, which is consistent with that of reported {001} facets [34]. Figure 4*f* shows a SAED image of ET-15. The spots were assigned to {101}, {004}, {200} and {105} faces, which was in agreement with the XRD results, verifying that the TiO_2_ attached to the surface of the RGO was in the anatase phase.

### 3.4. Raman analysis

**Figure 5.**
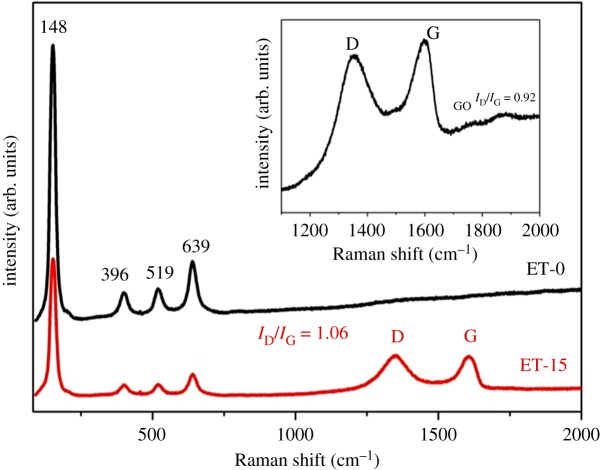
Raman spectra of GO, ET-0 and ET-15.

Raman spectroscopy is a powerful and widely employed technique for the characterization of *sp*^2^ and *sp*^3^ hybridized carbon atoms that are contained in graphene, which includes disordered and defect structures [28]. The Raman spectra of GO (inset), ET-0 and ET-15 are presented in figure 5. The peaks located at 148, 396, 519 and 639 cm^−1^ were assigned to anatase TiO_2_ [35]. Peaks at the same position could also be observed in ET-15; however, the intensities of the peaks were much weaker than those of ET-0. The reason is that strong diffraction peaks could be generated in ET-15 at higher positions following combination with graphene, which weakened the peak intensities at lower positions [36]. It was reported that there were two characteristic signals assigned to graphene in the Raman spectra, which were the G band at 1596 cm^−1^ and the D band at 1360 cm^−1^ [31,37]. The D band is common for disordered *sp*^2^ carbon, whereas the G band is also referred to as the E_2 g_ mode, which is observed in well-ordered graphite [30,31]. Of particular interest was the intensity ratio of the D and G bands, *I*_D_/*I*_G_, which is a measure of the relative concentration of local defects or disorder (particularly *sp*^3^ hybridized defects) relative to the *sp*^2^ hybridized graphene domains. These two bands could be found in the spectra of both GO and ET-15. Further, the *I*_D_/*I*_G_ ratio increased in ET-15 compared to that of GO, showing that the graphene was reduced in ET-15 [25,38].

### 3.5. X-ray photoelectron spectrophotometry results

**Figure 6.**
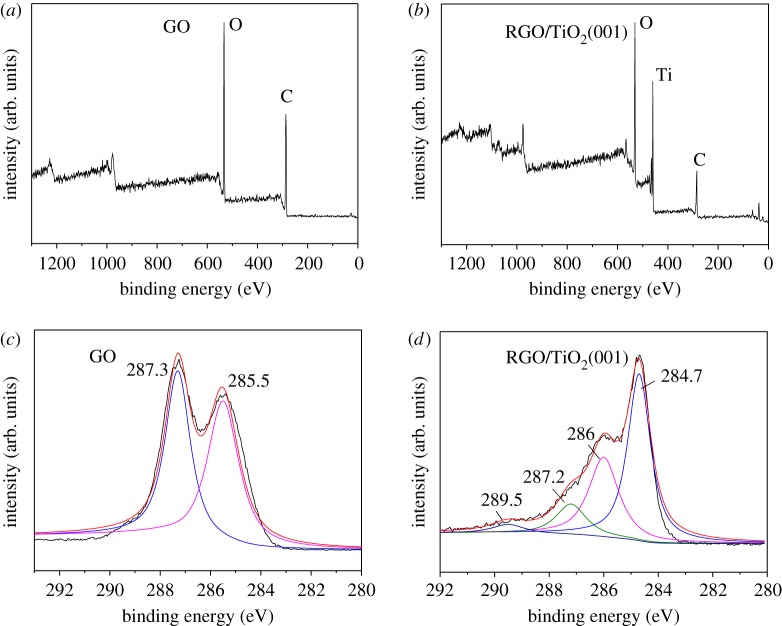
XPS spectra. Survey scans of GO (*a*) and RGO/TiO_2_ (001) (*b*). High-resolution spectra of C1s of GO (*c*) and RGO/TiO_2_ (001) (*d*).

The surface elements of GO and RGO/TiO_2_(001) were investigated via XPS with the results presented in figure 6. It can be seen from survey scan of figure 6*a* that the surface of GO was composed only of C and O elements. After combination with TiO_2_(001), GO was reduced into RGO. And the surface of RGO/TiO_2_(001) was composed of C, O and Ti elements (figure 6*b*). The high-resolution spectra of C1s in GO and RGO/TiO_2_(001) are displayed in figure 6*c,d*. Two typical C1s peaks of GO were observed at 285.5 and 287.3 eV, which are usually assigned to adventitious carbon and *sp*^2^-hybridized carbon from the GO, and the oxygen-containing carbonaceous bands (C–OH), respectively [30]. The C1s spectrum of RGO/TiO_2_(001) could be deconvoluted into four peaks with positions at 284.7, 286, 287.2 and 289.5 eV, respectively. The peak of 284.7 eV is assigned to the C–C, C=C and C–H bonds (*sp*^2^) of graphene [28]. The peaks at 286, 287.2 and 289.5 eV are attributed to the C–OH, C=O and O=C–OH oxygen-containing carbonaceous bands, respectively [28,31]. Such a surface functional group indicated that the –OH groups on the TiO_2_ possibly react with the –COOH groups on the GO surface through esterification to form O=C–O–Ti bonds [30]. In C1s spectra of pure GO, high amount of oxygen-containing functional groups were observed, which is consistent with pristine GO. And after combination with TiO_2_(001) during the hydrothermal process, the oxygen-containing functional groups decreased dramatically, indicating GO was reduced into RGO, which was in accordance with Raman results.

### 3.6. Photoluminescence analysis

**Figure 7.**
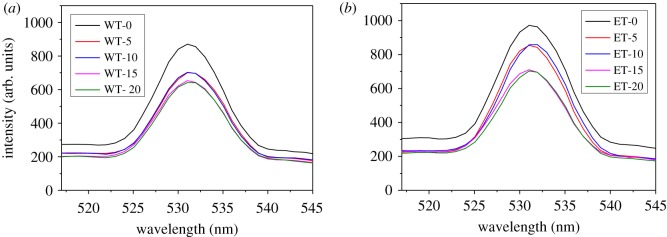
PL spectra of W (*a*) and E (*b*) catalysts.

When a semiconductor is excited with a light source that provides photons with energy larger than the bandgap energy, photo-induced electrons and holes are generated. When the photo-induced electrons and holes are recombined, luminescence is given out. Figure 7 shows the PL spectra of the materials. It can be seen that the PL intensities of the materials decreased dramatically after combination with graphene both in W and E catalysts, indicating that combination with graphene facilitated the charge transfer and thus inhibited PL intensities. Compared to WT-5 and WT-10, WT-15 and WT-20 exhibited even lower PL intensities. Similarly, in E catalysts, ET-15 and ET-20 exhibited weaker PL intensities than ET-5 and ET-10.

### 3.7. Photocatalytic activity

**Figure 8.**
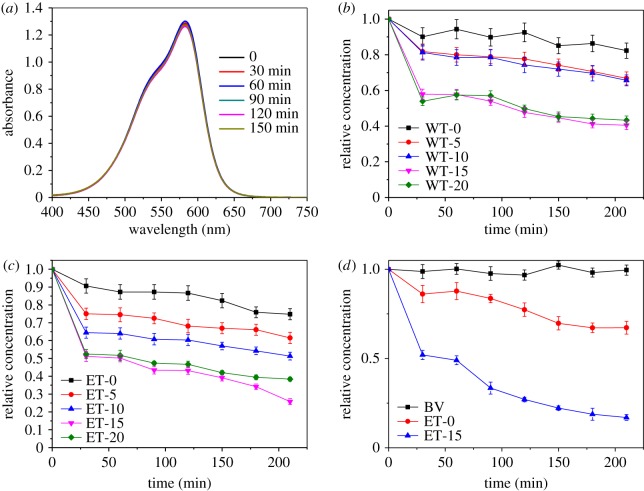
Degradation of BV under white light (*λ* > 390 nm, 5 W LED light) illumination without catalyst (*a*), with W catalysts (*b*), with E catalysts (*c*) and with E catalysts under visible light (*λ* = 420 nm, 6 W LED light) irradiation (*d*).

The photocatalytic activity of RGO/{001} faceted TiO_2_ was evaluated using BV as model pollutant under white light (*λ* > 390 nm, 5 W LED light) irradiation, with the results displayed in figure 8. Figure 8*a* shows the UV–visible absorption spectra of BV under white light irradiation without catalyst, which shows that the BV was stable and could hardly be degraded without a catalyst. The photocatalytic degradation of BV with W catalysts is presented in figure 8*b*. It may be seen that all of the RGO/{001} faceted TiO_2_ composites had higher photocatalytic efficiencies than that of pure {001} faceted TiO_2_, where the amount of RGO influenced the photocatalytic activities of the catalyst. The photocatalytic efficiency of the composites followed the trend as: WT-15 > WT-20 > WT-10 > WT-5 > WT-0. With WT-15, the BV was degraded by 60.2% in 2.5 h, which was 2.13 times that of WT-0.

The photocatalytic degradation of BV with the E catalysts is displayed in figure 8*c*. Similar to the W catalysts, all of the RGO containing materials exhibited higher photocatalytic activities than that of pure {001} faceted TiO_2_. Among all of the E catalysts, ET-15 demonstrated the highest photocatalytic activity. Over 2.5 h, the BV was degraded by 75.8 and 36.4% when catalysed by ET-15 and ET-0, respectively. Interestingly, the photocatalytic efficiency also followed the trend as: ET-15 > ET-20 > ET-10 > ET-5 > ET-0, which was consistent with W catalysts, showing that a 15% RGO content was the most appropriate for both W and E catalysts.

To further investigate the notion that the incorporation of RGO facilitates improvements in the activities of visible or UV light, the photocatalytic degradation of BV with ET-15 and ET-0 under visible light (*λ* = 420 nm, 6 W LED light with intensity of 1.45 × 10^5^ lux) irradiation was carried out, with the results displayed in figure 8*d*. It can be seen that BV could not be degraded under visible light irradiation without a catalyst in 2.5 h. In the photocatalytic processes, the BV was degraded by 83.6 and 33.5% with ET-15 and ET-0, respectively. That is to say, the photocatalytic efficiency of ET-15 was 2.5 times that of ET-0 under visible light irradiation (2.08 times under white light irradiation), which suggested that the combination with RGO improved visible light activity more efficiently.

**Figure 9.**
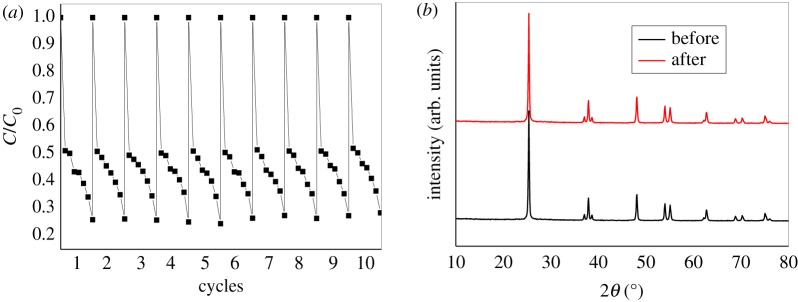
Recycling experiments with ET-15 under white light irradiation (*a*) and XRD patterns before and after 10 cycles.

To test the stability of as-prepared materials, recycling experiments with ET-15 under white light irradiation were conducted, and the results are shown in figure 9*a*. It can be seen that after 10 cycles running, the photocatalyst still retained high photocatalytic efficiency in BV degradation. The crystal structure of ET-15 after 10 cycles was characterized via XRD, all the diffraction peaks being consistent with those before recycle experiments (figure 9*b*), showing that as-prepared materials were very stable during the photocatalytic processes.

**Figure 10.**
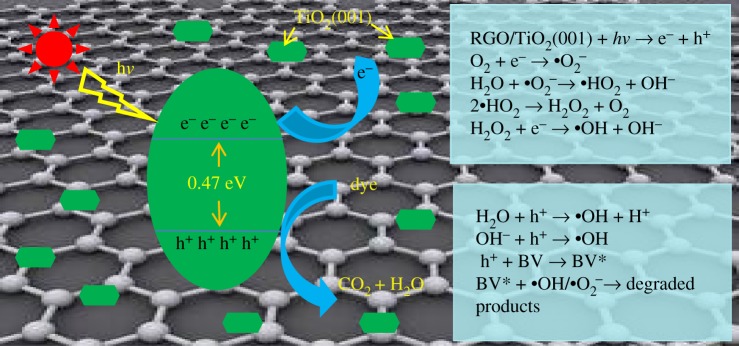
Illustration of photocatalytic process on RGO/TiO_2_ (001).

Gao *et al*. [39] explored the interfacial properties of hybrid graphene/anatase TiO_2_ (001) nanocomposites using first-principle calculations, which were based on density functional theory, and found that the band structure of the graphene/TiO_2_ (001) was modified, and the energy gap was reduced to 0.47 eV. The *E*_g_ of the graphene/TiO_2_ (001) was much lower than that of TiO_2_ (001), which consequently resulted in the electronic transition from valence bands (VB) to conduction bands (CB) in graphene/TiO_2_ (001) more easily than that in TiO_2_ (001). Furthermore, it initiated the absorption edge red shift to the longer wavelength region, as well as a striking difference in electron–hole pair formation. To be precise, the separation of electrons and holes in graphene/TiO_2_ (001) occurred more easily than that in TiO_2_ (001), indicating the photocatalytic performance of graphene/TiO_2_ (001) was more potent than that of TiO_2_ (001). Based on the experiments results and the theoretical calculation, the possible photocatalytic reaction mechanism was proposed and illustrated in figure 10. Under visible light irradiation, electrons are easily excited from VB to CB in graphene/TiO_2_ (001) and then transferred to graphene. Subsequently, •O_2_^−^ is generated by O_2_ and e^−^, and •HO_2_ or •OH are formed by •O_2_^−^ with H_2_O. On the other hand, h^+^ is gathered in the VB and may react with H_2_O to form •OH. As a result, the dye molecule is degraded into different products by the series of radicals generated above.

In addition, E catalysts had higher photocatalytic efficiencies compared to W catalysts, which was likely due to a more efficient combination between RGO and {001} faceted TiO_2_ caused by the addition of ethanol. This resulted in the more effective separation of photo-induced electrons and holes, as well as charge transfer, as graphene has been reported to be an important candidate for a charge acceptor due to its 2D planar-conjugation structure [29].

## 4. Conclusion

RGO/{001} faceted TiO_2_ was successfully synthesized via a facile solvothermal method using water and ethanol as regents. The E catalysts exhibited higher photocatalytic activities compared to W catalysts, due to the more efficient integration of graphene and {001} faceted TiO_2_ with the addition of ethanol during preparation process. Incorporation of graphene into {001} faceted TiO_2_ narrowed the bandgap greatly and enhanced the photocatalytic activity, due to the convenient transition of electrons from TiO_2_ to graphene, particularly under visible light irradiation. These composites are very stable and warrant further investigations towards promising applications in photocatalysis, water splitting, solar energy and green chemistry fields, in order to use visible light more efficiently.

## Data accessibility

This article has no additional data.
